# A review of swarm intelligence algorithms deployment for scheduling and optimization in cloud computing environments

**DOI:** 10.7717/peerj-cs.696

**Published:** 2021-08-25

**Authors:** Yousef Qawqzeh, Mafawez T. Alharbi, Ayman Jaradat, Khalid Nazim Abdul Sattar

**Affiliations:** 1 Department of Computer Science and Engineering, Hafr Al Batin University, Hafr AL Batin, Saudi Arabia; 2 Department of Natural and Applied Sciences, Buraydah Community College, Qassim University, Buraydeh, Qassim, Saudi Arabia; 3 Computer Science and Information Department, Majmaah University, AlZulfi, Riyadh, Saudi Arabia

**Keywords:** Swarm Intelligence, Optimization, Cloud Computing, Scheduling, Task-Allocation

## Abstract

**Background:**

This review focuses on reviewing the recent publications of swarm intelligence algorithms (particle swarm optimization (PSO), ant colony optimization (ACO), artificial bee colony (ABC), and the firefly algorithm (FA)) in scheduling and optimization problems. Swarm intelligence (SI) can be described as the intelligent behavior of natural living animals, fishes, and insects. In fact, it is based on agent groups or populations in which they have a reliable connection among them and with their environment. Inside such a group or population, each agent (member) performs according to certain rules that make it capable of maximizing the overall utility of that certain group or population. It can be described as a collective intelligence among self-organized members in certain group or population. In fact, biology inspired many researchers to mimic the behavior of certain natural swarms (birds, animals, or insects) to solve some computational problems effectively.

**Methodology:**

SI techniques were utilized in cloud computing environment seeking optimum scheduling strategies. Hence, the most recent publications (2015–2021) that belongs to SI algorithms are reviewed and summarized.

**Results:**

It is clear that the number of algorithms for cloud computing optimization is increasing rapidly. The number of PSO, ACO, ABC, and FA related journal papers has been visibility increased. However, it is noticeably that many recently emerging algorithms were emerged based on the amendment on the original SI algorithms especially the PSO algorithm.

**Conclusions:**

The major intention of this work is to motivate interested researchers to develop and innovate new SI-based solutions that can handle complex and multi-objective computational problems.

## Introduction

Scheduling and optimization techniques have been applied to cloud environments, leading to a research area called evolutionary scheduling and optimization and represents the integration of artificial intelligence (AI) and operational research. Working with a cloud environment is complex and tedious in that there is a large need for better scheduling and optimization strategies. Since the computational problems are generally multi-objective in nature and complex, the traditional scheduling and optimization techniques are considered inadequate. With the emergence of AI techniques, these AI-based algorithms boosted the performance of scheduling and optimization approaches. Over the years, it has become abundantly manifest that these biodiversity of resources strategies are great enhancer for cloud computing work environments; therefore, they provide a robust, reliable, and enhanced strategies for better task achievement and workload distribution among the available cloud resources.

**Figure 1:**
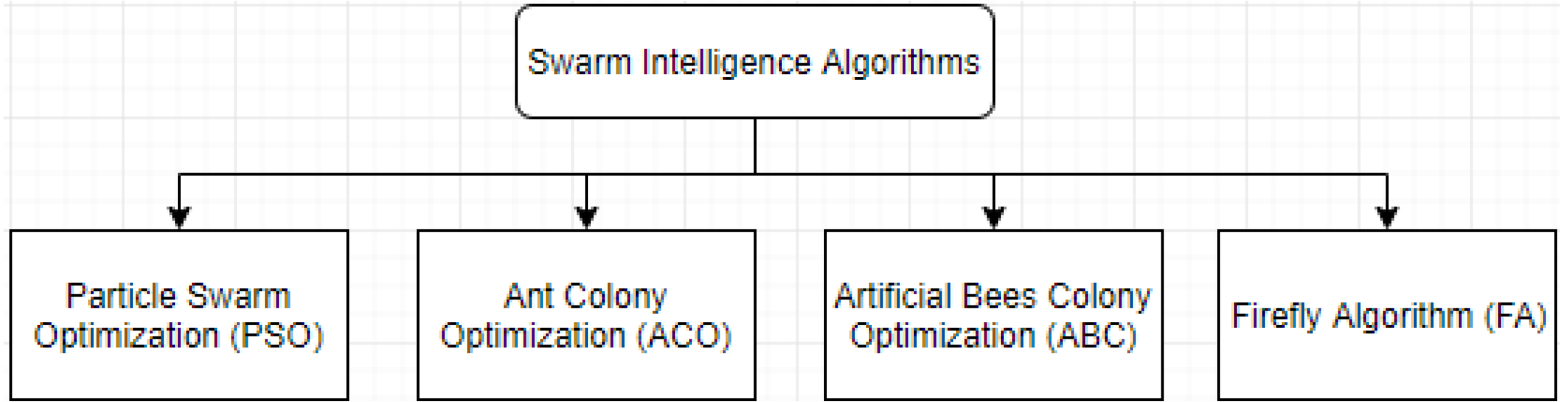
The reviewed SI-based algorithms hierarchy.

The evolutionary algorithms mimic species evolution based on Darwin’s theory. They form a cluster of algorithms in which the genetic algorithm (GA) was the first proposed one. Recently, several SI algorithms have been applied widely to solve complex multi-objective problems. The ant colony optimization (ACO), for example, has been utilized in weather routing and in the travelling salesman problem (TSP). The particle swarm optimization (PSO) algorithm had a large implementation in constrained and unconstrained functional optimization problems. However, this article aims to review and summarize the most recent publications of SI techniques. In cloud computing, task scheduling is very important (Zuo et al., 2015) due to its direct effect in the performance of systems. As task scheduling problems are considered NP-hard problems, they need to meet user needs and improve the overall performance of the systems. This work would be of interest to the students and readers in this domain since SI algorithms lack enough supporting publications and resources as compared to the well-known methods like neural networks or genetic algorithms. Moreover, the challenge of identifying the changes of parameter settings in the mentioned SI methods in this work, and the hybrid associations between the existing SI approaches, facilitates the way for students and readers in this domain better understand and build on the existing techniques. Figure 1 presents the reviewed SI-based algorithms in this work.

This review contributes to the overall deployment of SI based techniques namely PSO, ABC, ACO, and FA, respectively, in scheduling and optimization problems. As many researchers had emphasized the importance of hybrid swarms in solving multi-objective scheduling and optimization problems, this review provided a reference in which it summarizes the recent hybrid techniques used to solve multi-objective problems. The review also highlighted the importance of parameter settings in scheduling problems. Additionally, it elaborated on the basics of SI methods that focused on the implementation of PSO, ABC, ACO, and FA in cloud environment scenarios. However, this study is structured into different sections starting with a basic introductory background. This is followed by a survey methodology which is then followed by sections separately dealing with SI-based algorithms in details. The algorithms discussed among SI-based are PSO, ACO, ABC, and FA studies. A future directions of action discusses the probability of some algorithms to gain prominence in research. Finally, a conclusions section has been introduced.

### Survey methodology

This review is conducted in several stages. First of all, the particular algorithms to be investigated were identified. Second, the authors paid attention to the hybrid nature between the identified SI-algorithms and the commonly parametric settings for problem solving. The aim of this review was to survey the literature to determine how SI algorithms were modified and hybridized to improve the performance of scheduling in cloud computing environment. However, this review scoped the latest publications for the following algorithms namely (PSO, ACO, ABC, and FA) in scheduling and optimization problems. Therefore, the last 5 years’ publications for the mentioned algorithms were almost reviewed with a focus on determining the main parameters that have been changed or modified to optimize the performance of each SI algorithm in cloud computing environment. However, this review highlighted the hybrid approach between the reviewed algorithms. In addition, a summarized and brief applications for the identified algorithms were conducted. Finally, the review is unbiased since it just highlighted the main modified parameters in the above-mentioned SI algorithms and their applications in the last five years. It is noteworthy that other SI methods such as Cuckoo search and Levy flights were excluded from the current study, thereby decreasing the scope in order to have a detailed discussion.

### Particle Swarm Optimization (PSO)

The PSO represents a well-known metaheuristic optimization technique due to its ease implementation in unsupervised, and complex problems. It is a reliable technique that has been used for treating several optimization problems. In fact, it is based on a physical model in which its transition rules are constructed by mimicking the social collective behavior observed from flocks of birds and/ or schools of fish (Sengupta, Basak & Peters, 2018). The PSO initializes a swarm of particles in which they traverse the search space looking for an optimal global best. In fact, each particle represents a potential solution. Suppose that *X*_*i*(*t*)_ be the position of any *i*th particle at a given time t. Then, its velocity is regulated based on the current position being lower than the global best position (Sengupta, Basak & Peters, 2018). Table 1 illustrates the metaheuristic PSO algorithm.

**Table 1:** PSO metaheuristic algorithm.

1	Initialize particles population in hyperspace
2	While termination criteria not met do
3	Evaluate fitness of individual particles
4	Modify velocities based on previous best and global best
5	End-While

**Figure 2:**
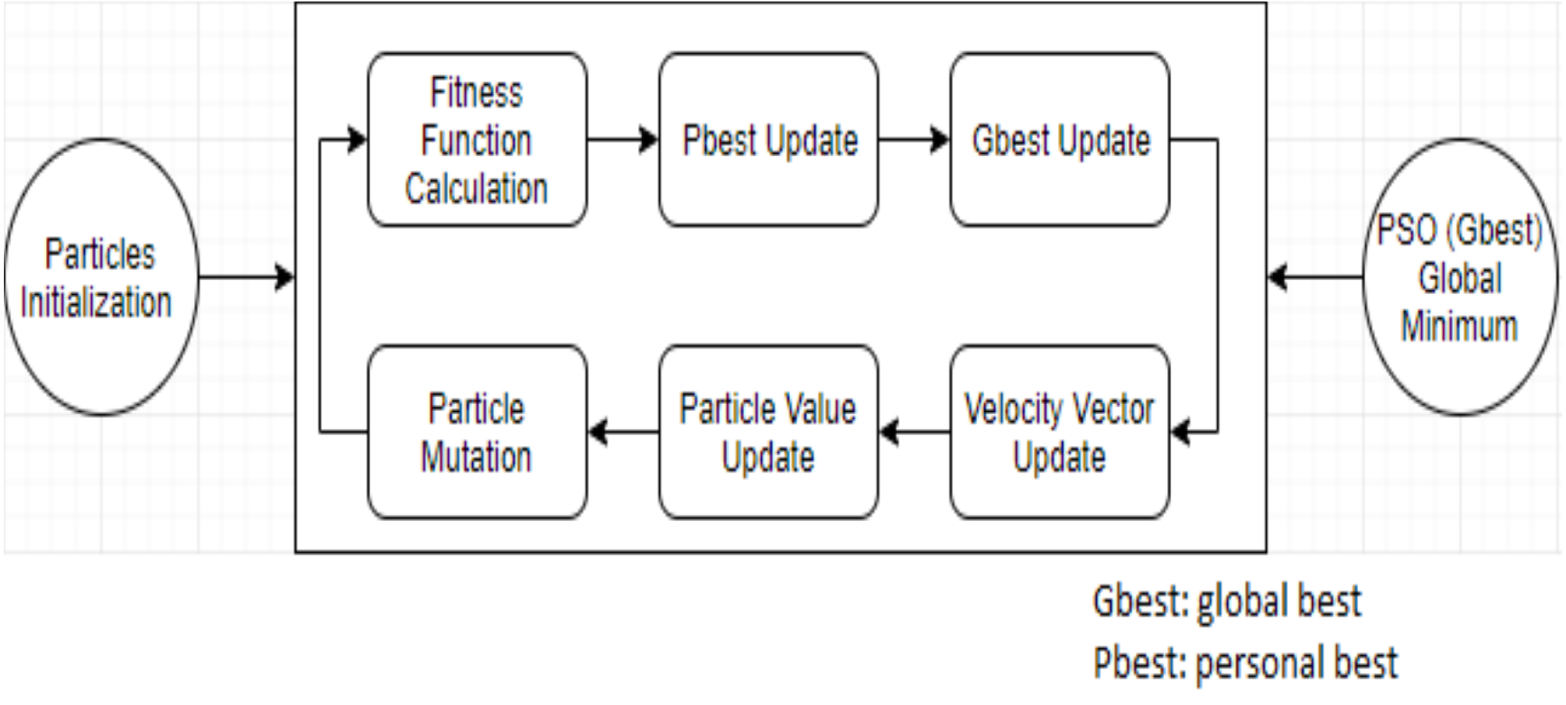
The original PSO algorithm.

As the particles search for food sources using original PSO, they may stick in local search. This may lead to less convergence in the search space. Due to some major shortcomings of the original PSO algorithm such as stagnation and local optima convergence, several studies were conducted to overcome such flaws. For these reasons, several researchers proposed a modified PSO or hybrid PSO computational strategies to enhance the performance of the original PSO algorithm. Generally, any given optimization problem concerns the selection of best possible solution that minimizes or maximizes a utility function based on some constraints. Therefore, SI-based approaches could introduce an acceptable and reliable solution to scheduling and optimization problems. SI-based solution for any given optimization problem, can be though as watching a team of players who cooperate together, share information, and update their positions collectively seeking a goal that formulates the rapprochement of victory (global best solution). Hence, seeking an efficient task allocation, a hybrid GA-PSO algorithm was proposed by Manasrah & Ali (2018). The proposed GA-PSO aimed at the reduction of the following parameters: makespan, cost, and load balance of cloud computing dependent tasks. However, another study by Ebadifard & Babamir (2018) presented a static task scheduling approach to improve PSO performance by utilizing a load balancing method. Another study by Alsaidy, Abbood & Sahib (2020) tried to improve PSO’s initialization using both “longest job to fastest processor (LJFP)” and “minimum completion time (MCT)” methods. Additionally, Zhou et al. (2018) proposed a cloud computing model based on energy consumption named M-PSO that is capable of handling the slow convergence issue and local optimum. Tabrizchi, Kuchaki Rafsanjani & Balas (2021) proposed a self-adaptive hybrid method named ICA-PSO to handle the multi-tasking scheduling issue by combining PSO and imperialist competitive algorithm (ICA) algorithms. Using reverse learning and gene mutation methods, Li et al. (2021) proposed a modified PSO to improve population diversity. To minimize the total execution time, a hybrid scheduling approach named GA-PSO was proposed by Senthil Kumar, Parthiban & Siva Shankar (2019) using PSO and genetic algorithm (GA). Moreover, to allocate tasks to a computing resources efficiently, Alkhashai & Omara (2016) presented two hybrid algorithms named Best-Fit-PSO (BFPSO) and PSO-Tabu Search (PSOTS). Another modified PSO named LBMPSO was introduced by Pradhan & Bisoy (2020) to schedule resources in cloud computing environment based on load balancing. Liu et al. (2019) proposed an adaptive disruption algorithm to enhance global and local search. Moreover, Farid et al. (2020) presented a survey on PSO to assist researchers and users determine the most important QoS considerations in cloud computing environments. The literature claimed that many researchers tried to develop new PSO scheduling strategies to enhance optimal solution convergence by introducing different load balancing methods. However, vectors in PSO can be updated using Eqs. (1), (2) below. Figure 2 illustrates the basic PSO algorithm.

(1) \begin{eqnarray*}{V}_{ij}^{t+1}=w{V}_{ij}^{t}+c1{r}_{1}^{t} \left( pbes{t}_{ij}-{X}_{ij}^{t} \right) +c2{r}_{2}^{t} \left( gbes{t}_{j}-{X}_{ij}^{t} \right) \ldots \ldots \ldots \ldots \ldots \ldots \end{eqnarray*}

(2) \begin{eqnarray*}{X}_{ij}^{t+1}={X}_{ij}^{t}+{V}_{ij}^{t+1}\ldots \ldots \ldots \ldots \ldots \ldots \end{eqnarray*}

### Ant Colony Optimization (ACO)

Ant colony optimization algorithm has a great advantage in addressing the combinatorial optimization problems. Several studies had investigated the scheduling tasks using ant colony algorithm in cloud computing environment. They normally categorized according to their targets focus such as scheduling efficiency, system performance, or cost. ACO represents an intelligent algorithm for path planning (Dai et al., 2019; Jovanovic, Tuba & Voß, 2016; Wang, Lin & Wang, 2016). It has a strong calculative mechanism (Ahmed et al., 2020). Generally, it is used for optimization by updating the pheromone trails and orienting the ants around the search space by which each ant generates a new fitness function to be used for generating an overall global fitness. The next state in ACO strategy is determined by the roulette wheel method in which it will be repeated till the goal point has been achieved. Upon the completion of each iteration, the ants update the pheromone trails along the length of path planning. In the available literature, the ACO has been applied mostly to society detection with single objective (Shahabi Sani, Manthouri & Farivar, 2020), while it has been applied to a multi-objective ACO optimization using decomposition (Mu et al., 2019). In fact, ant colony inspired researchers in how ants find the best route to food source. Updating the pheromone trails is proposed by Ekmekci (2019) using a modified ACO that memorizes solution costs.

**Figure 3:**
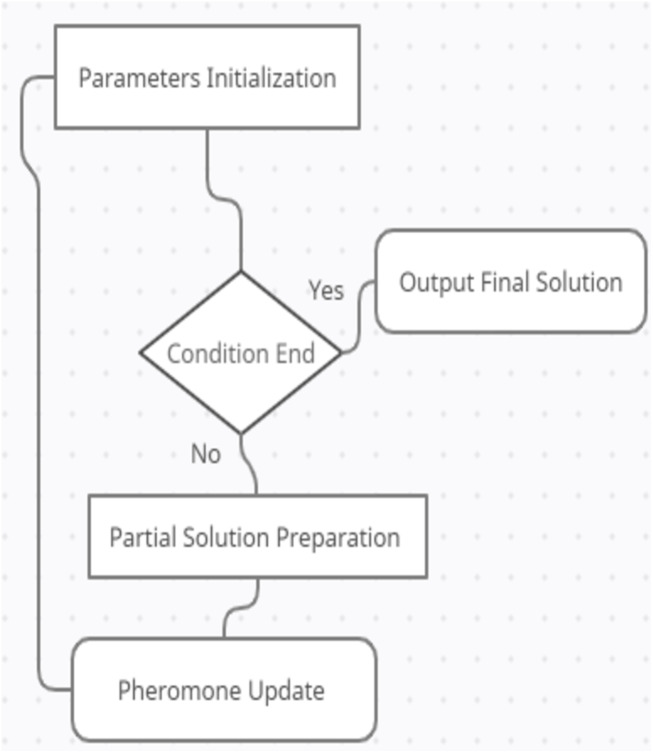
The ACO Diagram.

To solve the TSP problem, Tamura, Sakiyama & Arizono (2021) introduced a modified ACO algorithm using individual memories (IM) named Ant System Using Individual Memories (ASIM) that aims to optimize ant’s diversity in the search space. Seeking an optimal solution for ship-weather routing multi-objective optimization problem, Zhang et al. (2021) introduced an improved ACO algorithm considering several parameters such as fuel consumption, sailing time, and navigation safety. A unified adaptive ACO algorithm was proposed by Yuan, Yuan & Huang (2017) related to SNP epistasis with multi-objective functions detection GWAS datasets. In addition, Senthil Kumar & Venkatesan (2019) proposed a hybrid genetic-PSO (HGPSO) algorithm to solve the problem of task scheduling. Another modified PSO algorithm named IPSO was introduced by Yu (2020) to optimize resource scheduling efficiency. The generated solution around the gbest for each ant can be expressed as in Eq. (3). The ACO algorithm diagram can be seen in Fig. 3.

(3) \begin{eqnarray*}{Z}_{i}^{d}=N \left( gbes{t}_{i}^{d},{\sigma }_{i} \right) \ldots \ldots \ldots \ldots \ldots \end{eqnarray*}

### Artificial Bee Colony Optimization (ABC)

The ABC represents a meta-heuristic approach for investigating the behavior of bees. A typical ABC’s applications can be found in several areas such as cloud computing, image processing, big data analysis, and neural networks. The ABC algorithm represents the most successful optimization SI algorithms (Aslan & Karaboga, 2020). They introduced a new ABC strategy for big data optimization using several signal decomposition techniques. Many studies (Wang et al., 2019) (Dokeroglu, Sevinc & Cosar, 2019) (Ranjan & Krishna, 2018) integrated both ABS and PSO algorithms seeking some sort of optimization in terms of updating personal best and global best objective functions. Seeking better random-initial allocation solutions to the scout bees in the ABC optimization, Beed, Roy & Bhattacharya (2019) proposed a hybrid PSO-Bees algorithm to solve multi-objective optimization problems. Another hybrid ABC-Heuristic technique was proposed by Kruekaew & Kimpan (2020) to improve scheduling solutions in virtual machines in homogeneous and heterogeneous cloud computing environments. A new added control-mechanism to the original ABC algorithm was introduced by Aslan (2019) to model the transitions of the employed-bees into dancing area. Trying to solve “Job-Shop-Scheduling-Problem (JSSP)” by minimizing the make-span, a new discrete ABC algorithm named DABC was introduced by Witkowski (2019). As computer-computing speed develops, many SI techniques are utilized in crowed evacuation research (Zhao et al., 2020). A multi-strategy ABC algorithm was introduced by Xiang et al. (2019) to enhance the comprehensive performance of the original ABC algorithm using a neighborhood search method. A study by Sheoran, Mittal & Gelbukh (2020) used the ABC strategy in the data-flow testing field to prioritize the definition-use paths. Moreover, a modified ABC algorithm was proposed by Sharma, Sharma & Sharma (2018) to solve the job-shop scheduling problem (JSSP). However, food source initialization and food source updating strategy of ABC algorithm are described in Eqs. (4), (5) below.

(4) \begin{eqnarray*}{X}_{ij}={X}_{i}^{min}+rand \left( 0,1 \right) \left( {X}_{j}^{max}-{X}_{j}^{min} \right) \ldots \ldots \ldots \ldots \ldots \ldots \ldots \end{eqnarray*}

(5) \begin{eqnarray*}{V}_{ij}={X}_{ij}+\varnothing \left( {X}_{ij}-{X}_{ki} \right) \ldots \ldots \ldots \ldots \ldots \ldots \ldots \end{eqnarray*}

### Firefly Algorithm (FA)

The firefly algorithm (FA) represents a metaheuristic approach that mimics the flashing behavior of fireflies (Farahlina Johari et al., 2017). It represents an evolutionary optimization approach. It has been applied to various challenging applications (Xia et al., 2018). Nayak et al. (2020) conducted an in-depth study about the variants, importance, and applications of FA in biomedical engineering (BME) fields. However, a comparison concerns the performance of PSO and firefly algorithm has been presented by Windarto & Eridani (2020) focusing on parameter estimation of “Lotka–Volterra” type competition model. Their comparison was based on profit data of rural bank and commercial bank in Indonesia. Moreover, to enhance the quality of the solution of “Unrelated parallel machine scheduling problem (UPMSP)”, a modified salp swarm algorithm (SSA) based on FA was proposed by Ewees, Al-qaness & Elaziz (2021) using the operators of FA to enhance SSA’s exploitation capability for working as a local search. Seeking an optimum machining parameter such as feed rate, spindle speed, and depth of cut, a hybridized strategy based on FA and PSO was developed by Farahlina Johari et al. (2017) to achieve an improved solution for search space exploration. To decrease time complexity of the original FA algorithm, Umbarkar, Balande & Seth (2017) proposed a modified FA algorithm that ranks the fireflies based on a quick sort algorithm instead of bubble sort technique.

**Figure 4:**
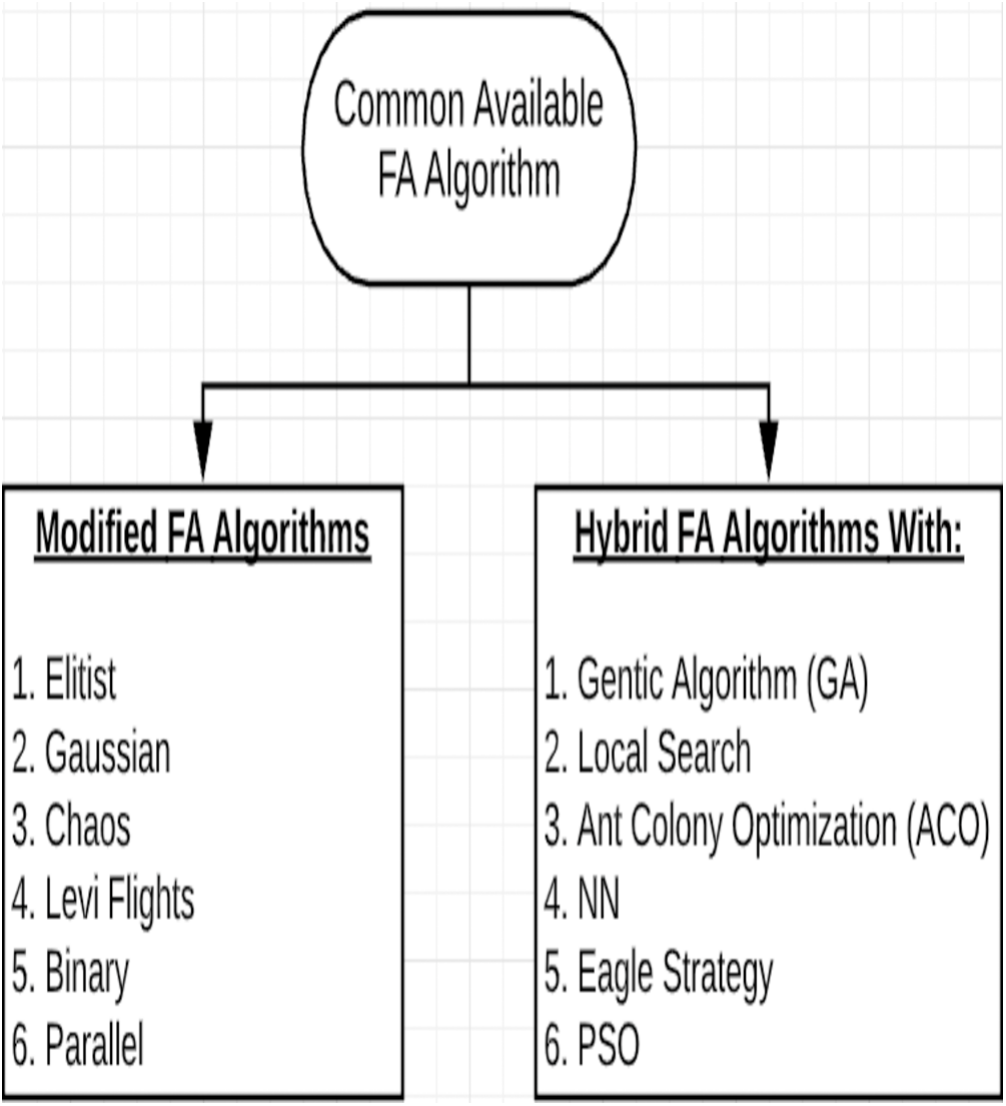
Commonly available FA algorithms.

To handle the optimal operation of thermal generating unit’s problem, an improved FA algorithm named improved firefly algorithm (IFA) was proposed by Nguyen, Quynh & Van Dai (2018) to reduce the cost of electricity generation fuel. Another study by Ma et al. (2019) aimed to overcome the problem of FA being fall into slow convergence and local extremum, by using a reverse learning initialization and Levy perturbation mechanisms based on FA algorithm. A hybrid optimizer based on PSO and FA entitled “FAPSO” has been proposed by Xia et al. (2018) to sanction the flies to explore more favorable sub-regions. Another study by Tighzert, Fonlupt & Mendil (2019) tried to reduce the computational cost and memory storage of the traditional FA algorithm by introducing a compact firefly approach that uses a minimum computational cost. Wahid & Ghazali (2019) proposed pattern search (PS) to terminate the FA to handle the drawback of the standard FA in its ending phase as it fails to get the optimal value since there is no observed improvement to the quality of the outcomes. A study by Hussein & Jaber (2020) introduced a modified FA algorithm to handle unit commitment problems. They claimed that the modified FA algorithm is more efficient than the classical FA in the selection of generator and error amid load and generation. To solve several non-linear “convex” optimal power flow (OPF), a hybrid algorithm consists of FA and PSO named “HFPSO” algorithm was introduced by Khan et al. (2020). Additionally, Dai, Liang & Zhang (2020) proposed a new FA-based algorithm to search for the optimal solution for uplift effect in the high-pressure jet grouting (HPJG) project. Another study (Peng et al., 2018) proposed a hybrid fish swarm algorithm based on the behavior of Lévy flight and firefly named LFFSA to overcome the problem of local optimum convergence. Several components (logistic and Gauss, Lévy flight, and adaptive inertia weight) were utilized by Chou & Ngo (2017) to introduce a modified FA algorithm (MFA) to optimize the multidimensional structural design. To adjust the parameters of the proportional integral-derivative (PID) controller in a buck converter, a hybridized algorithm using FA and PSO named HFPSO was proposed by Ekinci et al. (2019). Figure 4 represents the common modified and hybrid FA algorithms.

V.1 FA Position Update

In FA, the distance between any two fireflies *i* and *j* at *X*_*i*_ and *X*_*j*_ can be calculate using Cartesian distance as per Eq. (6).

(6) \begin{eqnarray*}{r}_{ij}= \left\vert {X}_{i}-{X}_{j} \right\vert =\sqrt{\sum _{k=1}^{d}({X}_{i}k-{X}_{j}k)^{2}\ldots \ldots \ldots \ldots \ldots \ldots \ldots }\end{eqnarray*}

*where k represents spatial coordinate component andd represents the number of dimensions*.

However, the movement of any firefly *i* towards an attractive firefly *j* can be described as per Eq. (7) below.

(7) \begin{eqnarray*}{X}_{i}={X}_{i}+{\beta }_{0}{e}^{-\gamma r2} \left( {X}_{j}-{X}_{i} \right) +\alpha \left( rand-0.5 \right) \ldots \ldots \ldots \ldots \ldots \ldots \ldots \end{eqnarray*}

*where *α* randomization parameter and r and is function generator*.

**Table 2:** The modified swarm intelligence methods and their applications.

***#***	**Title**	**Authors**	**Problem to be solved**	**The proposed solution**
**PSO Algorithm**				
1	Multi-task Scheduling Algorithm Based on Self-adaptive Hybrid ICA–PSO Algorithm in Cloud Environment	Tabrizchi, Kuchaki Rafsanjani & Balas (2021)	Multi-tasking scheduling issue	Self-adaptive hybrid PSO named ICA-PSO
2	A Modified PSO Algorithm for Task Scheduling Optimization in Cloud Computing	Zhou et al. (2018)	Slow convergence issue and local optimum	Modified PSO named “M-PSO”
3	A Novel Load Balancing Technique for Cloud Computing Platform Based on PSO	Pradhan & Bisoy (2020)	Load balancing rescheduling	LBMPSO
4	BF-PSO-TS: Hybrid Heuristic Algorithms for Optimizing Task Schedulingon Cloud Computing Environment	Alkhashai & Omara (2016)	Inefficient tasks allocation	Best-Fit-PSO (BFPSO) and PSO-Tabu Search (PSOTS)
5	A PSO-Based Task Scheduling Algorithm Improved Using a Load-Balancing Technique for the Cloud Computing Environment	Ebadifard & Babamir (2018)	Low performance of PSO	Static task scheduling using load balancing
6	A modified particle swarm optimization for large-scale numerical optimizations and engineering design problems	Liu et al. (2019)	Large-scale numerical optimizations and engineering design problems	Cauchy mutation technique
**ACO Algorithm**				
7	An Ant Colony Optimization Memorizing Better Solutions (ACO-MBS) for Traveling Salesman Problem	Ekmekci (2019)	Tuning the pheromone trail	Memorizes the solution costs and updates the pheromone trail
8	Mobile Robot Path Planning Based on Ant Colony Algorithm With A* Heuristic Method	Dai et al. (2019)	Low convergence and deadlock problem	Using of A ∗ algorithm ubrk and MAX-MIN Ant system to improve ACO heuristics.
9	Multi-Objective Ant Colony Optimization Algorithm Based on Decomposition for Community Detection in Complex Networks	Mu et al. (2019)	Multi-objective optimization	A modified ACO algorithm
10	Application of Improved Multi-Objective Ant ColonyOptimization Algorithm in Ship Weather Routing	Zhang et al. (2021)	Ship-weather routing optimization problem	Using of modified ACO
11	Ant Colony Optimization Using Common Social Information and Self-Memory	Tamura, Sakiyama & Arizono (2021)	Traveling salesman problem (TSP)	ASIM (Modified ACO algorithm using individual memories (IM))
**ABC Algorithm**				
12	A genetic Artificial Bee Colony algorithm for signal reconstruction based big data optimization	Aslan & Karaboga (2020)	Big data optimization	Using of several signal decomposition techniques
13	Particle swarm optimization and discrete artificial bee colony algorithms for solving production scheduling problems	Witkowski (2019)	Job-Shop-Scheduling-Problem (JSSP)	Discrete ABC algorithm named DABC
14	FAACOSE: A fast adaptive ant colony optimization algorithm for detecting SNP Epistasis	Yuan, Yuan & Huang (2017)	SNP epistasis detection	A unified adaptive ACO algorithm
15	An improved artificial bee colony algorithm for pavementresurfacing problem	Ranjan & Krishna (2018)	pavement resurfacing optimization problem	ABC algorithm for eliminating thetrigger roughness level specification beforehand.
16	Beer froth artificial bee colony algorithm for job-shop scheduling problem	Sharma, Sharma & Sharma (2018)	JSSP	Modified BeFABC algorithm for JSSP
**FA Algorithm**				
17	Enhanced Salp Swarm Algorithm Based on Firefly Algorithm for Unrelated Parallel Machine Scheduling with Setup Times	Ewees, Al-qaness & Elaziz (2021)	UPMSP	modified salap algorithm (SSA) based on FA
18	Unit Commitment Based on Modified Firefly Algorithm	Hussein & Jaber (2020)	Unit commitment problems	Modified FA algorithm
19	Firefly Optimization Algorithm for the Prediction of Uplift Due to High-Pressure Jet Grouting	Dai, Liang & Zhang (2020)	HPJG	FA algorithm with Stochastic medium theory (SMT)
20	Modified Firefly Algorithm for Multidimensional Optimization in Structural Design Problems	Chou & Ngo (2017)	Multidimensional structural design	A modified FA algorithm (MFA)
21	Hybrid Firefly and Particle Swarm Optimization Algorithm for PID Controller Design of Buck Converter	Ekinci et al. (2019)	Proportional integral-derivative (PID) controller adjustment	A hybridized algorithm using FA and PSO (HFPSO)

### The future research directions of SI algorithms

This review explored the potential applications of PSO, ABC, ACO, and FA algorithms. However, there was a little research on multi-objective scheduling and optimization compared to a single-objective approach. This indeed highlights the importance of utilizing the swarm algorithms in solving complex multi-objective problems. As many scholars developed a modified SI algorithm, Table 2 summarizes the modified SI methods and their applications. It can be utilized to further introduce new modified or hybrid algorithms and to be used for multi-objective complex problems optimization. PSO algorithm, for instance, still has a potential for more optimized modifications in which it can be self-adaptive to handle loss of diversity, and/ or local optima stagnation issues. Moreover, despite the modifications that carried out on ACO algorithm, still it can be improved for applications that are more practical. Expert prior knowledge can also be applied to ACO algorithm in future, which believed to improve efficiency of ACO algorithm. Additionally, ABC can be applied to parallel computation in run-time by tuning its parameters in which this could optimize solutions to the NP-Hard combinatorial problems. Path prioritization represents a promising challenge for ABC algorithm. However, there are several areas in which FA algorithm can be utilized to cope with. Applying FA’s variations such as Gaussian, and/ or multi-population in biomedical engineering (BME) and healthcare (HC) areas can be fruitful research opportunities. Table 3 illustrates the most used parameters for modification. However, the main challenges in SI-based in scheduling and optimization problems are multi-objective search, multidimensional numeric problems, fitness function improvement, local optima escaping, global optima finding. Figure 5 shows a count analysis for the reviewed articles in this survey.

**Table 3:** Most modified swarms’ parameters.

#	Parameter	Usage
1	PSO (Inertia weight)	To control the swarm velocity
2	PSO (Acceleration coefficients)	Enhance efficiency and stability
3	ACO (α)	To determines the influence of the pheromone trail
4	ACO (β)	To determine heuristic value
5	ABC (Scout bees)	To balance exploration vs exploitationTo balance exploration vs exploitation
6	ABC (Tabu List size)	
7	FA (β0)	Initial attractiveness
8	FA (γ)	Absorption parameter

**Figure 5:**
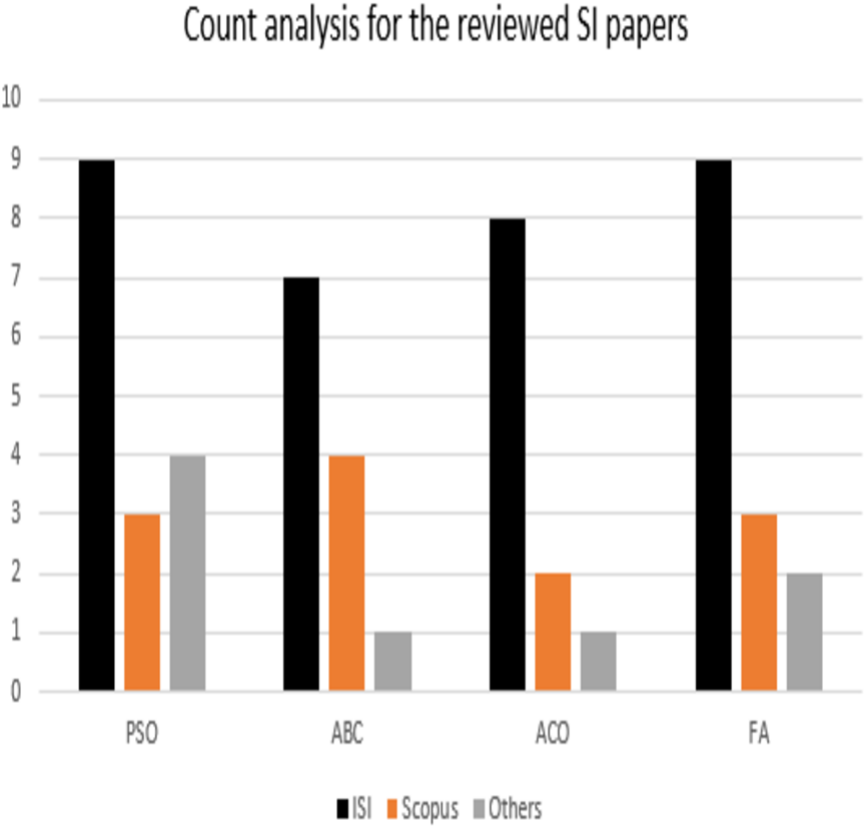
Count analysis for the reviewed articles in this survey.

## Conclusions

This review focused on the overall deployment of SI-based algorithms (PSO, ABC, ACO, and FA, respectively) in scheduling and optimization problems. As stated in the literature, several scholars tried to introduce new SI-based scheduling strategies to optimize search space exploration and exploitation by proposing a variety of parametric-modification and load balancing schemes. However, many researchers had emphasized the importance of hybrid swarms in solving multi-objective scheduling and optimization problems. As task-scheduling represents a major issue in cloud computing environment, many hybrid techniques were proposed by scholars to improve the performance of traditional SI algorithms and to allocate the suitable cloud resources to user tasks. However, the reviewed algorithms in general lose solution quality when their dimensionality increased. It is noteworthy that parameter settings for one problem do not operate for every problem. However, the basics of SI methods that target the implementation and illustration of PSO, ABC, ACO, and FA in cloud environment scenarios have been elaborated in detail. In addition, some of the most current and noteworthy applications of SI-based techniques for cloud environment scheduling we are surveyed. The future plan for this work aims to develop a hybrid SI strategy utilizing the most dominant parameters such as inertia weight, acceleration coefficients, and Tabu list size.

## Additional Information and Declarations

### Data Availability

The following information was supplied regarding data availability: This review work has no raw data or code to be submitted.
